# Evaluating Individual Level Responses to Exercise for Health Outcomes in Overweight or Obese Adults

**DOI:** 10.3389/fphys.2019.01401

**Published:** 2019-11-14

**Authors:** Leanna M. Ross, Cris A. Slentz, William E. Kraus

**Affiliations:** ^1^Duke University Medical Center, Duke Molecular Physiology Institute, Durham, NC, United States; ^2^Division of Cardiology, School of Medicine, Duke University, Durham, NC, United States; ^3^Urbaniak Sports Sciences Institute, School of Medicine, Duke University, Durham, NC, United States

**Keywords:** exercise prescription, lifestyle medicine, individual variation, cardiovascular health, precision medicine, training response

## Abstract

**Background:**

Understanding group responses to a given exercise exposure is becoming better developed; however, understanding of individual responses to specific exercise exposures is significantly underdeveloped and must advance before personalized exercise medicine can become a functional reality. Herein, utilizing data from the STRRIDE studies, we address some of the key issues surrounding our efforts to develop better understanding of individual exercise responsiveness.

**Methods:**

We assessed individual cardiometabolic and cardiorespiratory fitness responses in subjects successfully completing STRRIDE I (*n* = 227) and STRRIDE II (*n* = 155). Subjects were previously sedentary, overweight or obese men and women with mild-to-moderate dyslipidemia. Subjects were randomized to either an inactive control group or to an exercise training program. Training groups varied to test the differential effects of exercise amount, intensity, and mode on cardiometabolic health outcomes. Measures included fasting plasma glucose, insulin, and lipids; blood pressure, minimal waist circumference, visceral adipose tissue, and peak VO_2_. Absolute change scores were calculated for each subject as post-intervention minus pre-intervention values in order to evaluate the heterogeneity of health factor responsiveness to exercise training.

**Results:**

For subjects completing one of the aerobic training programs, change in peak VO_2_ ranged from a loss of 37% to a gain of 77%. When ranked by magnitude of change, we observed discordant responses among changes in peak VO_2_ with changes in visceral adipose tissue, HDL-C, triglycerides, and fasting plasma insulin. There was also not a clear, direct relationship observed between magnitudes of individual response in the aforementioned variables with aerobic training adherence levels. This same pattern of highly variable and discordant responses was displayed even when considering subjects with adherence levels greater than 70%.

**Conclusion:**

Our findings illustrate the unclear relationship between magnitude of individual response for a given outcome with training adherence and specific exercise exposure. These discordant and heterogeneous responses highlight the difficult nature of developing understanding for how individuals will respond to any given exposure. Further investigation into the biological, physiological, and genetics factors affecting individual responsiveness is vital to making personalized exercise medicine a reality.

## Introduction

Understanding the benefits of exercise at a group or population level has several purposes. At a population level – for the purposes of public health – understanding group responses and the variability around these responses provides information about cost-benefit and what health messages and initiatives should be provided to a population so as to be able to obtain the greatest benefit for the population as a whole. This is the goal of the recently released 2018 Physical Activity Guidelines for Americans (U.S Department of Health and Human Services, 2018) and the science that supports these guidelines (Physical Activity Guidelines Advisory and Committee, 2018).

Understanding the benefits of exercise at the individual level has other purposes. Assessing the heterogeneity of exercise training effects on health benefits at an individual level and being able to predict what benefits will accrue to an individual in response to a given exercise regimen may allow one to prescribe a specific program for a specific health benefit to accomplish one of the main goals of personalized exercise medicine – the right exercise regimen, for the right condition, for the right person, at the right time (Kraus, 2017). However, to use physical activity or exercise prescriptions to reduce risk for disease or treat a developing health problem, one needs to be able to predict with some precision what magnitude of response in health parameters one might expect to see at an individual level from any one of a number of different exercise regimens. We are now just beginning to develop these understandings to be able to approach this question with some precision; nonetheless, there is still some way to go (Buford et al., 2013; Ozemek and Arena, 2019).

Both intensity and amount effects of aerobic exercise contribute to improvements in cardiorespiratory fitness and in the various components of the metabolic syndrome (Duscha et al., 2005; Johnson et al., 2007; Bateman et al., 2011). At the group level, greater amounts of aerobic exercise drive improvements in HDL-cholesterol (Kraus et al., 2002), waist circumference (Slentz et al., 2004) and visceral adiposity (Slentz et al., 2005); and intensity of aerobic exercise drives reductions in fasting insulin and triglycerides in a paradoxical fashion, whereby moderate intensity has a more beneficial effect than vigorous intensity when the same amount (energy expenditure) of exercise is performed (Kraus et al., 2002; Houmard et al., 2004). As the relationship of various characteristics of exercise to health improvements is multifaceted at the group level, the heterogeneity of response at the individual level introduces even greater complexity. Within an individual, the magnitude of favorable or adverse change in any given health parameter may bear an unpredictable relationship to favorable or adverse responses to the same exercise regimen for other equally important health parameters. The purpose of this paper is to discuss these concepts using a secondary analysis of data from the STRRIDE studies (Kraus et al., 2001; Slentz et al., 2011), including our understanding of the challenges and limitations of a personalized approach to lifestyle medicine as they currently exist.

## Materials and Methods

We assessed the heterogeneity of cardiometabolic and cardiorespiratory fitness responses in data from STRRIDE I (Kraus et al., 2001) and II (Slentz et al., 2011). STRRIDE I (1999–2003) and STRRIDE II (2004–2008) enrolled previously sedentary, overweight or obese men and women with mild-to-moderate dyslipidemia (classified by low-density lipoprotein-cholesterol (LDL-C): 130–190 mg/dL or high-density lipoprotein cholesterol (HDL-C): ≤40 mg/dL for men and ≤45 mg/dL for women). In STRRIDE I, subjects were randomized into one of four groups: (1) inactive control; (2) low amount/moderate intensity aerobic exercise: 14 kcal of energy expenditure/kg of body weight/week (KKW) at 40–55% peak oxygen consumption (VO_2_); (3) low amount/vigorous intensity aerobic exercise: 14 KKW at 65–80% peak VO_2_; (4) high amount/vigorous intensity aerobic exercise: 23 KKW at 65–80% peak VO_2_. Of the 334 subjects randomized, 227 subjects successfully completed STRRIDE I (32.0% drop out rate). In STRRIDE II, subjects were randomized to one of four groups: (1) low amount/vigorous intensity aerobic exercise: 14 KKW at 65–80% peak VO_2_; (2) high amount/vigorous intensity aerobic exercise: 23 KKW at 65–80% peak VO_2_; (3) resistance training only: 3 days/week, 8 exercises, 3 sets/exercise, 8–12 repetitions/set; (4) linear combination of the low amount/vigorous intensity aerobic and resistance training programs. Of the 210 subjects randomized in STRRIDE II, 155 subjects successfully completed the study (26.2% drop out rate). Both STRRIDE I and II study protocols were approved by the institutional review boards at Duke University and East Carolina University. Subjects provided both verbal and signed written informed consent.

### Laboratory Measurements

Subjects underwent multiple laboratory measures at baseline and post-intervention. Body mass and height were assessed while subjects were wearing light clothing and no shoes. Blood pressure was measured at rest in a seated position. Waist circumference was measured at the minimal waist, which is the least circumference measurement obtained above the umbilicus and below the xiphoid (Willis et al., 2007). Computed tomography scans were performed by a radiological technologist and the images were analyzed using Slice-o-matic imaging software from Tomo Vision to determine the area of visceral adipose tissue (Slentz et al., 2005).

Fasting blood samples were obtained from the beginning of an intravenous glucose tolerance test at baseline and 16–24 h after the final exercise bout. Fasting plasma glucose was determined via a YSI analyzer (Yellow Springs, OH, United States), and HDL-C as well as triglycerides were determined via nuclear magnetic resonance spectroscopy (LipoScience, Raleigh, NC, United States). Fasting plasma insulin was determined via immunoassay (Access Immunoassay System, Beckman Coulter, Fullerton, CA, United States).

Subjects completed graded maximal cardiopulmonary exercise tests on a treadmill with 12-lead electrocardiography and expired gas analysis (TrueMax Parvomedics; Provo, UT, United States). Within STRRIDE I and STRRIDE II, the same study staff members performed all tests. The graded treadmill test protocol consisted of 2-min stages, starting at 3 mph and 0% grade, then increased speed and/or grade by approximately one metabolic equivalent per stage until the subject reached volitional exhaustion. The two greatest, consecutive 15-s readings were averaged to determine peak VO_2_.

### Exercise Training

For the aerobic training groups, subjects underwent an initial ramp period of 2–3 months in order to allow subjects to gradually adapt to their exercise prescription. The ramp period was followed by six additional months of training at the appropriate exercise prescription. The intensity of prescribed exercise was based on the individual baseline cardiopulmonary exercise test results. Aerobic exercise modes included treadmills, elliptical trainers, cycle ergometers, or any combination of these.

For the resistance training groups, the ramp period started with one set during weeks 1–2, two sets during weeks 3–4, and built up to the three set prescription on week 5. The prescription included three sessions per week (non-consecutive days), of three sets of 8–12 repetitions on eight Cybex weight-lifting machines. The resistance exercises were designed to target all major muscle groups. To ensure a progressive resistance training stimulus throughout the intervention, the amount of weight lifted was increased by 2.75 kg each time the subject performed 12 repetitions properly on all three sets during two consecutive workout sessions.

For the aerobic training groups, exercise intensity and duration for all exercise sessions were verified by direct supervision and/or with the use of downloadable heart rate monitors (Polar Electro, Woodbury, NY, United States). Aerobic training adherence was calculated for each subject as the number of minutes completed within the prescribed heart rate range, divided by the number of total weekly minutes prescribed. Resistance training sessions were verified by direct supervision and/or the FitLinxx Strength Training Partner (FitLinxx, Norwalk, CT, United States). The “training partner” automatically sent data from each session to the FitLinxx server computer. The computers recorded total weight lifted via laser weight plate detection, and total number of repetitions (which were only counted when subjects lifted through the full range of motion), and finally speed of weight lifting motion was monitored and alerts were given when subjects lifted too quickly.

### Statistical Analyses

To further illustrate the heterogeneity of cardiometabolic response to exercise intervention, we assessed both group and individual level changes in the metabolic syndrome *z*-score in the STRRIDE I cohort. As described in a previous STRRIDE study (Johnson et al., 2007), the metabolic syndrome *z-*score was used as a continuous score of the five metabolic syndrome variables. A modified *z*-score was calculated for each metabolic syndrome variable using individual subject data, the Adult Treatment Panel (ATP) III criteria (Grundy et al., 2005), and baseline standard deviations for the entire STRRIDE I cohort. To account for variations in ATP III criteria between men and women, we used sex-specific metabolic syndrome *z*-score equations. For women, metabolic syndrome *z*-score = [(50 − HDL-C)/14.1] + [(triglycerides − 150)/81.0] + [(fasting plasma glucose − 100) /11.3] + [(waist circumference − 88)/9.0] + [(mean arterial pressure − 100)/9.1]. For men, metabolic syndrome *z*-score = [(40 − HDL-C)/9.0] + [(triglycerides − 150)/81.0] + [(fasting plasma glucose − 100)/11.3] + [(waist circumference − 102)/7.7] + [(mean arterial pressure − 100)/9.1].

For all outcome variables, absolute change scores were calculated for each subject as the difference between the post-minus pre-intervention values. We also calculated percent change for peak VO_2_ as the absolute change score divided by the pre-intervention value. At the group level, paired *t* tests were performed to determine significant mean changes within groups; a *p*-value of 0.05 was used to indicate statistical significance (STATVIEW, SAS, Cary, NC, United States). To illustrate the individual variability of responses to exercise training, waterfall plots were created for peak VO_2_ and metabolic syndrome *z*-score, in which each bar represents an individual subject’s change score.

## Results

Overall, subjects completing STRRIDE I (*n* = 227; 46.7% female; 80.6% Caucasian) were 52.4 ± 6.4 years old and had a body mass index of 29.7 ± 3.0 kg/m^2^ at baseline. STRRIDE I graduates also had an average baseline peak VO_2_ of 27.8 ± 5.9 mL/kg/min, mean systolic blood pressure of 128.9 ± 14.8 mmHg, mean diastolic blood pressure of 83.3 ± 8.0 mmHg, minimal waist circumference of 95.2 ± 9.7 cm, HDL-C of 45.9 ± 13.8 mg/dL, triglycerides of 152.5 ± 82.5 mg/dL, and fasting plasma glucose of 93.0 ± 10.2 mg/dL.

At baseline, STRRIDE II graduates (*n* = 155; 55.5% female; 84.5% Caucasian) were 48.9 ± 10.2 years old and had a body mass index of 30.5 ± 3.3 kg/m^2^. In addition, STRRIDE II graduates had an average peak VO_2_ of 27.4 ± 6.0 mL/kg/min, mean systolic blood pressure of 119.3 ± 13.6 mmHg, mean diastolic blood pressure of 78.1 ± 8.9 mmHg, minimal waist circumference of 96.4 ± 9.5 cm, HDL-C of 45.3 ± 12.2 mg/dL, triglycerides of 139.7 ± 71.0 mg/dL, and fasting plasma glucose of 96.4 ± 11.8 mg/dL at baseline.

**FIGURE 1:**
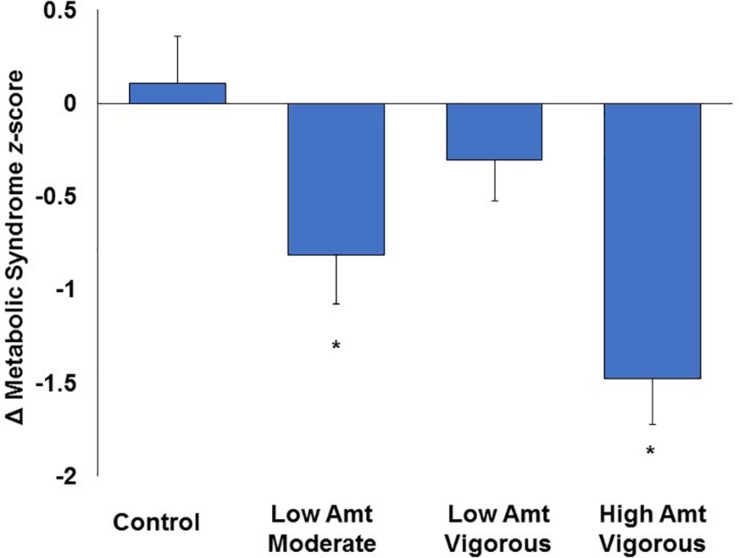
Effects of amount and intensity of exercise on changes in metabolic syndrome *z*-score at the group level in STRRIDE I. Amt, amount; ^∗^ represents *p* < 0.05 for within-group change with exercise training. Reprinted from Johnson et al. (2007) with permission from Elsevier, Copyright 2007.

**FIGURE 2:**
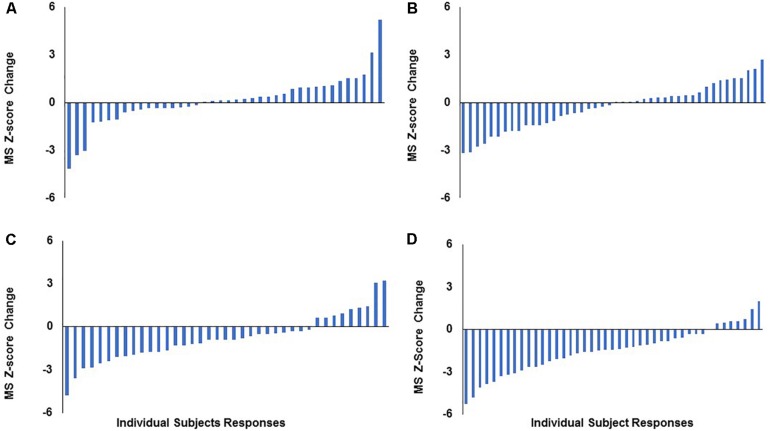
Distribution of individual responses for change in metabolic syndrome *z*-score following 8 months of aerobic exercise training in STRRIDE I. Each bar represents an individual subject’s change score. **(A)** Control group; **(B)** low-amount/vigorous-intensity group; **(C)** low-amount/moderate-intensity group; **(D)** high-amount/vigorous-intensity group. MS, metabolic syndrome.

When assessing mean change at the group level in STRRIDE I (Johnson et al., 2007), both the low amount/moderate intensity and high amount/vigorous intensity groups significantly decreased their metabolic syndrome *z*-scores following 8 months of exercise training, while the inactive control and low amount/vigorous intensity groups did not significantly change compared to baseline (Figure 1). However, when plotting individual change scores ordered by magnitude of change, all four groups display a broad range of responses, from negative to positive (Figure 2). As shown in Figure 2D, even the group with the greatest average improvement in metabolic syndrome *z*-score (−1.5 ± 1.6 points for the high amount/vigorous intensity group) included individuals who either had no significant improvement or even an increase in their metabolic syndrome *z*-score.

**FIGURE 3:**
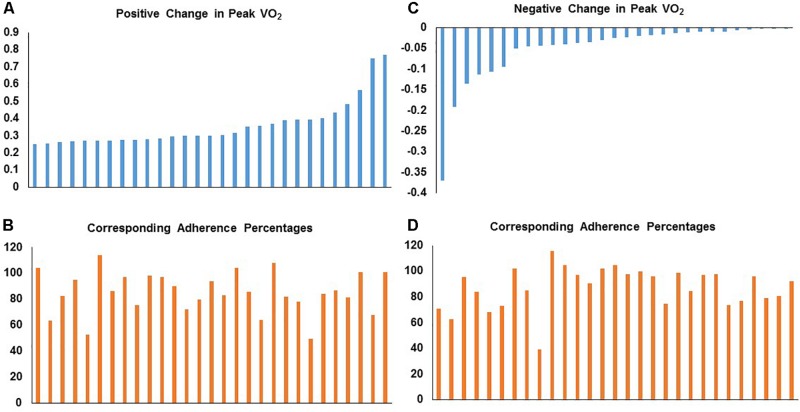
Distribution of individual responses for fractional change in peak VO_2_ with corresponding aerobic training adherence (percent) in STRRIDE I and STRRIDE II. Each bar represents an individual subject’s change score or adherence level. Panel **(A)** represents individuals with the greatest positive change in peak VO_2_ with their corresponding training adherence in panel **(B)**. Panel **(C)** represents individuals with the greatest negative to no response in peak VO_2_ with the corresponding training adherence in panel **(D)**.

We also evaluated the heterogeneity of responsiveness of peak VO_2_ in both STRRIDE I and STRRIDE II. By order of magnitude, we ranked all graduates who participated in an aerobic training group by percent change peak VO_2_. Across both cohorts, change in peak VO_2_ ranged from a loss of 37.0% to a gain of 76.8%. We plotted these individual change scores for those with the greatest positive change in peak VO_2_ (ranging from 25.2 to 76.8%; *n* = 29) with the corresponding training adherence in Figures 3A,B. We also plotted the individual change scores for the 29 subjects with the greatest negative to no response in peak VO_2_ (ranging from −37.0 to −0.2%) and their corresponding training adherence (Figures 3C,D). To further explore the substantial variability in peak VO_2_ response and confirm that the subjects performed ideal maximal exercise tests, we evaluated the peak respiratory exchange ratios (RER) achieved during the exercise tests to serve as an objective measure of maximal effort. For the 58 subjects listed in Figure 3, we found that pre-intervention peak RER values averaged 1.16 and the post-intervention peak RER values averaged 1.13. In addition, only five individual tests out of 116 tests had RER’s below 1.0.

In addition, we assessed these 58 subjects with the most negative and positive peak VO_2_ responses by their corresponding changes in the following cardiometabolic risk factors: visceral adipose tissue, HDL-C, triglycerides, and fasting plasma insulin. In both tables, change scores that were greater than the technical variation in the test in the favorable direction are shaded green. Change scores greater than the technical variation in an unfavorable direction are shaded in red. As illustrated in Tables 1, 2, there was discordance in response across all variables within individual subjects. For any given group of outcome measures, subjects having a negative response in one outcome variable did not necessarily have a negative response in the other risk factors. Taking together, the illustrations in Figure 3 and Tables 1, 2, there does not appear to be a clear, direct relationship between magnitude of individual response for a given outcome variable with aerobic training adherence nor specific training stimulus group. Even when considering only the subjects who had aerobic training adherence levels greater than 70%, the same pattern is observed: large heterogeneity and discordance of responses across a variety of important cardiometabolic health risk factors.

**TABLE 1:** Non-cordance of responses – negative peak VO_2_ change.

**Percentchangein**	**Training group**	**Aerobic training**	**Delta VAT**	**Delta HDL-C**	**Delta TG**	**Delta FI**
**peak VO_2_ (%)**		**adherence (%)**	**(cm^2^)**	**(mg/dL)**	**(mg/dL)**	**(uU/mL)**
−37.0	Vig/Low	71		2.9	35.9	6.0
–19.2	Mod/Low	63		8.7	–15.4	–0.7
–13.6	Vig/Low/Resistance	96	–89	10.2	–39.8	–4.1
–11.4	Vig/Low/Resistance	84		6.4	–10.6	–3.7
–10.7	Mod/Low	68	–19			0.2
–9.5	Mod/Low	73		–3.0	48.0	1.5
–5.0	Mod/Low	102	5	–1.6	3.5	–1.5
–4.6	Vig/Low	85	13	–0.3	–1.0	–5.0
–4.4	Vig/High	40	–14	0.2	15.3	–0.1
–4.2	Vig/Low	116	–23	13.2	–65.3	–1.3
–4.0	Mod/Low	105	–48	–2.0	4.0	–3.4
–3.7	Vig/Low	97	–53	17.1	106.7	–4.9
–3.5	Vig/Low	91	–46	–0.4	–31.2	–5.2
–3.1	Mod/Low	102	–69	3.1	–20.0	–6.7
–2.6	Mod/Low	105	–69	–5.8	–66.0	–3.7
–2.4	Mod/Low	98	3	–11.5	–24.0	–3.4
–2.0	Mod/Low	100	–21	–1.0	–224.0	–4.8
–1.8	Vig/Low	96	–4	2.9	–118.1	–1.3
–1.7	Mod/Low	75		–1.0	15.0	–4.1
–1.3	Mod/Low	99	23	–1.5	–38.0	–1.9
–1.2	Vig/Low/Resistance	85		–3.1	–64.0	–1.2
–1.1	Mod/Low	97	–11	–1.1	38.0	1.2
–1.0	Mod/Low	98	5	9.0	–50.0	–0.2
–0.9	Vig/Low/Resistance	74	40	12.6	37.2	–1.3
–0.6	Vig/High	77	–7	–3.9	–10.0	0.3
–0.5	Mod/Low	96	10	0.8	54.0	0.4
–0.3	Mod/Low	79		1.5	–15.2	–2.7
–0.3	Mod/Low	81		–1.0	101.0	
–0.2	Mod/Low	92	–1	–11.0	91.0	–1.2
0.6	Mod/Low	84	–1	–2.5	–30.7	–3.7
0.6	Mod/Low	85	–72	–7.0	–52.0	7.7
0.6	Mod/Low	101		3.7	–31.0	–2.0
0.7	Mod/Low	104		4.0	1.0	–0.3
0.8	Mod/Low	71	–5	–2.0	–30.2	–11.9

*Each row represents an individual subject’s data; Vig/Low, vigorous intensity and low amount of aerobic exercise training; Mod/Low, moderate intensity and low amount; Vig/Low/Resistance, vigorous intensity and low amount aerobic plus resistance exercise training; Vig/High, vigorous intensity and high amount; Delta VAT, change in visceral adipose tissue; Delta HDL–C, change in high-density lipoprotein-cholesterol; Delta TG, change in triglycerides; Delta FI, change in fasting insulin.*

**TABLE 2:** Non-cordance of responses – positive peak VO_2_ change.

**Percentchangein**	**Training group**	**Aerobic training**	**Delta VAT**	**Delta HDL-C**	**Delta TG**	**Delta FI**
**peak VO_2_ (%)**		**adherence (%)**	**(cm^2^)**	**(mg/dL)**	**(mg/dL)**	**(uU/mL)**
76.8	Mod/Low	101	9	–19.0	37.0	–0.6
74.7	Vig/Low/Resistance	68		–2.0	–14.1	
56.7	Vig/High	101	–42			–10.4
48.2	Vig/Low/Resistance	81	18	8.2	45.9	–6.7
43.6	Vig/Low	87	–6	1.9	19.1	–7.0
40.0	Vig/High	84		5.0	–8.0	–3.7
39.5	Vig/High	49		2.6	–42.4	–2.9
39.3	Vig/Low	78		10.5	13.0	1.3
39.0	Vig/High	82	–49	2.0	–10.0	1.7
37.0	Mod/Low	108	40			
35.5	Vig/High	64	21	–2.1	–21.7	0.3
35.1	Vig/Low/Resistance	86	44	4.3	–86.8	–5.1
31.4	Vig/Low	104	–99	7.8	–154.4	–9.2
30.2	Mod/Low	83		2.1	–20.6	1.0
30.1	Vig/High	94	–1	1.4	12.6	–3.6
29.8	Vig/High	80	5	1.0	–12.0	0.8
29.8	Vig/High	72		–5.9	7.0	0.6
29.7	Vig/High	90	30	6.5	57.9	5.7
28.3	Vig/High	97	–18	12.0	–23.0	–0.4
27.8	Mod/Low	98	–30	5.6	–81.0	3.6
27.5	Vig/Low/Resistance	76	–48	7.7	–8.5	–6.5
27.3	Vig/High	97	–58	5.0	–118.0	–0.3
27.2	Vig/Low/Resistance	57	–39	–2.9	–24.6	2.2
27.2	Mod/Low	114	–1	10.5	–27.4	–1.1
27.0	Vig/High	53		–2.2	12.7	2.7
26.6	Vig/High	95	–61	6.6	–13.1	2.3
26.1	Vig/High	83		5.0	–36.0	–3.4
25.5	Mod/Low	64		7.7	–104.0	–2.5
25.2	Mod/Low	104	–34	–4.6	–143.7	–0.3
24.7	Mod/Low	103	–29	–6.2	–68.3	0.4
24.3	Vig/High	65	–39	9.6	8.0	0.0
23.9	Mod/Low	111	–25	2.1	–92.9	0.1
23.5	Mod/Low	71	4			2.4
23.3	Mod/Low	87	–6			–0.9
23.2	Vig/Low	96	–38			

*Each row represents an individual subject’s data; Vig/Low, vigorous intensity and low amount of aerobic exercise training; Mod/Low, moderate intensity and low amount; Vig/Low/Resistance, vigorous intensity and low amount aerobic plus resistance exercise training; Vig/High, vigorous intensity and high amount; Delta VAT, change in visceral adipose tissue; Delta HDL-C, change in high-density lipoprotein-cholesterol; Delta TG, change in triglycerides; Delta FI, change in fasting insulin.*

## Discussion

From our work in STRRIDE, we have several key observations bearing on the issues surrounding our efforts to develop better understanding of individual exercise responsiveness. First, hidden beneath the well-defined health benefits of exercise at the group level reported in exercise training studies – where groups may be defined as different modes, intensities, or amounts of exercise exposure – there is significant heterogeneity of response at an individual level.

Second, this heterogeneity of response is defined by the error bars routinely reported in our study figures (e.g., Figure 1). The heterogeneity of the response clearly can be appreciated in a series of waterfall plots of response by group (see Figure 2 for metabolic syndrome *z*-score). The fact that this heterogeneity is intrinsic to the individual biology or physiology of an individual, and not a reflection of exposure, can be seen when the individual responses are plotted over the individual intervention adherence metrics (Figure 3 for change in cardiorespiratory fitness, peak VO_2_). Interestingly, even when we assessed the subjects with aerobic training adherence levels greater than 70%, we observed large variability and discordant responses across peak VO_2_, visceral adipose tissue, HDL-C, triglycerides, and fasting insulin (Tables 1, 2). At least part of the individual biology responsible for the individual heterogeneity of response is genetic (Sarzynski et al., 2016, 2017; Ross et al., 2019). In the case of change in cardiorespiratory fitness in response to aerobic exercise, approximately 50% is genetic (Timmons et al., 2010; Bouchard, 2012). Work is just now beginning to define the genetic predictors of response to other elements of the metabolic syndrome (Sarzynski et al., 2016). That said, because they do not yet replicate well across study populations, polygenic predictors of response for even change in peak VO_2_ to exercise are not well developed enough to be useful clinically (Timmons et al., 2010). In fact, this is true for even the strongest polygenic risk predictors for obesity and cardiovascular disease, where the predictive capacity for the best polygenic risk for obesity predicts a mere 29.2% of cases (Emdin et al., 2017; Khera et al., 2019).

Third, this heterogeneity is expected in continuous variables and is characteristic of a Gaussian distribution of responses across a population. The tails of this distribution are defined as being outside two standard deviations of the mean response (positive or negative), where 5% of the population will be at the tails of the response. When this extreme distribution is wide, and the lower tail crosses the technical variation of the health outcome variable, it can be termed an adverse or negative response (Bouchard et al., 2012). When the intervention exposure is modest or weak, even a greater number may be characterized as non-responders; however, this is more a characteristic of the exposure, not the individual. As an example, a recent study evaluated individual patterns of fitness response in a randomized, cross-over study in 21 healthy young adults comparing two different exercise exposures: 3 weeks of endurance cycling versus 3 weeks of sprint interval cycling (Bonafiglia et al., 2016). Both training protocols elicited similar main group effects, while the individual responses varied widely and the patterns of individual response differed by protocol. Although the study assessed the initial response to short-term training, the results support the hypothesis that individuals who do not favorably respond to a given exercise exposure may undergo more beneficial fitness responses to a different exercise exposure. However, we do not know whether these results would extend to longer duration training interventions and populations with additional risk factors, like the present study includes.

Fourth, the response of a given health parameter to a given exercise regimen for any given individual does not predict the response to a related health variable to the same intervention. As illustrated in Tables 1, 2, there is little correlation between cardiorespiratory fitness responses and some elements of the metabolic syndrome. For example, a high responder in cardiorespiratory fitness does not necessarily mean any given individual will be a high responder in metabolic syndrome or the constituent elements thereof (HDL-C, triglycerides, fasting insulin or visceral adiposity; Table 2). Similarly, a high responder in one element of the metabolic syndrome (e.g., HDL-C) is not necessarily a high responder, or responder at all, in the other elements of the metabolic syndrome.

Fifth, there is heterogeneity in the health outcome responses to the components of exercise exposure (intensity, amount, frequency, and mode). At the group level, both intensity and amount influence improvements in cardiorespiratory fitness in response to aerobic exercise training, while intensity influences responses in fasting insulin, triglycerides, and metabolic syndrome score in an opposite direction to that of how it influences cardiorespiratory fitness: greater intensity when controlling for amount leads to greater improvements in cardiorespiratory fitness; whereas, the converse is true (less intense exercise training controlling for amount) for fasting insulin, triglycerides, and metabolic syndrome score at a group level (Figure 1).

Sixth, in addition to the biological and physical contributors to the heterogeneity of the response of health outcomes to exercise training, there are biological – in fact, genetic – predictors of exercise and physical activity, even in humans (de Geus et al., 2014). The identification of such genetic variants may promote better targeted and individualized messaging for individuals less likely from a constitutional standpoint to engage in favorable physical activity behaviors. For instance, using a candidate gene approach, we identified a genetic variant in the *acid ceramidase* gene predicting non-completion – or drop-out – from our STRRIDE studies (Lewis et al., 2018). As has been demonstrated for other lifestyle behaviors such as smoking and nutrition (e.g., coffee consumption) (Cornelis et al., 2011; Gustavsson et al., 2012; Shenker et al., 2013; Matoba et al., 2019), this result provides evidence for the presence of biological predictors of exercise adherence. We are now underway in attempts to identify other genetic variants predicting exercise behavior in human STRRIDE subjects using a genome-wide approach.

### Limitations

We recognize some limitations of our study. In STRRIDE I and II, subjects were allowed to perform aerobic training on a combination of aerobic equipment, while exercise testing occurred solely on a treadmill. We recognize that cross-training effects may have occurred, which can lead to bias in the exercise testing results; however, we believe the aerobic training stimuli from treadmill and elliptical trainer use, which were the primary modes of aerobic training, should yield similar peak VO_2_ responses. We also recognize that the STRRIDE studies were not designed to specifically investigate individual-level exercise training responsiveness. Thus, the current paper is a secondary analysis highlighting the observed heterogeneity of responses.

## Conclusion

There are inherent individual biological, physiological, and genetics factors involved in determining the responses in a myriad of health parameters to exercise training of a given mode, intensity and amount. Indeed, not only is there heterogeneity at the individual level to exercise-induced health responses and behaviors, there is also heterogeneity at a group level for specific health parameters to exercise programs that differ in mode, intensity and amount. All of this heterogeneity makes predicting, at an individual level, favorable responses to exercise training inherently difficult, but not impossible. Developing more information in this arena may eventually make personalized exercise medicine a reality, permitting us not only to suggest exercise programs of a given mode, intensity or amount for a particular health condition, but also to target intensive behavioral reinforcement strategies for exercise to those most likely to be poorly adherent, or who avoid exercise altogether.

## Data Availability Statement

The datasets analyzed in this manuscript are not publicly available. Requests to access the datasets should be directed to william.kraus@duke.edu.

## Ethics Statement

The studies involving human participants were reviewed and approved by Duke University and ECU Institutional Review Boards. The patients/participants provided their written informed consent to participate in this study.

## Author Contributions

LR conceived the project, developed the data, and assisted in writing the manuscript. CS collected the data, synthesized the data, and assisted in editing the manuscript. WK conceived the project, obtained funding for the project, collected the data, and wrote the manuscript. All authors contributed to data analysis, interpretation of results, figure preparation, drafting and editing the manuscript, and approved the final version of the manuscript.

## Conflict of Interest

The authors declare that the research was conducted in the absence of any commercial or financial relationships that could be construed as a potential conflict of interest.
